# An *In-Silico* Corrosion Model for Biomedical Applications for Coupling With *In-Vitro* Biocompatibility Tests for Estimation of Long-Term Effects

**DOI:** 10.3389/fbioe.2021.718026

**Published:** 2021-09-07

**Authors:** Tijana Šušteršič, Gorkem Muttalip Simsek, Guney Guven Yapici, Milica Nikolić, Radun Vulović, Nenad Filipovic, Nihal Engin Vrana

**Affiliations:** ^1^ Faculty of Engineering, University of Kragujevac (FINK), Kragujevac, Serbia; ^2^ Steinbeis Advanced Risk Technologies Institute Doo Kragujevac (SARTIK), Kragujevac, Serbia; ^3^ Bioengineering Research and Development Center (BioIRC), Kragujevac, Serbia; ^4^ Mechanical Engineering Department, Faculty of Engineering, Ozyegin University, Istanbul, Turkey; ^5^ Institute of Information Technologies, University of Kragujevac, Kragujevac, Serbia; ^6^ Eindhoven University of Technology, Eindhoven, Netherlands; ^7^ SPARTHA Medical, Strasbourg, France

**Keywords:** in silico modelling, numerical simulations, biomaterial corrosion, cellular automata, implant surfaces, nickel titanium alloy

## Abstract

The release of metal particles and ions due to wear and corrosion is one of the main underlying reasons for the long-term complications of implantable metallic implants. The rather short-term focus of the established *in-vitro* biocompatibility tests cannot take into account such effects. Corrosion behavior of metallic implants mostly investigated in *in-vitro* body-like environments for long time periods and their coupling with long-term *in-vitro* experiments are not practical. Mathematical modeling and modeling the corrosion mechanisms of metals and alloys is receiving a considerable attention to make predictions in particular for long term applications by decreasing the required experimental duration. By using such *in-silico* approaches, the corrosion conditions for later stages can be mimicked immediately in i*n-vitro* experiments. For this end, we have developed a mathematical model for multi-pit corrosion based on Cellular Automata (CA). The model consists of two sub-models, corrosion initialization and corrosion progression, each driven by a set of rules. The model takes into account several environmental factors (pH, temperature, potential difference, etc.), as well as stochastic component, present in phenomena such as corrosion. The selection of NiTi was based on the risk of Ni release from the implant surface as it leads to immune reactions. We have also performed experiments with Nickel Titanium (NiTi) shape memory alloys. The images both from simulation and experiments can be analyzed using a set of statistical methods, also investigated in this paper (mean corrosion, standard deviation, entropy etc.). For more widespread implementation, both simulation model, as well as analysis of output images are implemented as a web tool. Described methodology could be applied to any metal provided that the parameters for the model are available. Such tool can help biomedical researchers to test their new metallic implant systems at different time points with respect to ion release and corrosion and couple the obtained information directly with *in-vitro* tests.

## Introduction

Main metallic biomaterials such as stainless steels, cobalt-chromium based alloys, medical grade titanium, Ti6Al4V and nickel-titanium shape memory alloys are well established biomaterials due to their strength, superior corrosion resistance and biocompatibility. However, immune reaction to such metals, particularly in long-term, after nanoparticle presence due to wear and also corrosion in the highly abrasive environment of body fluids can create chronic inflammation related complications. Nickel Titanium shape memory alloys with their shape memory effect and super elasticity are well positioned for applications where actuation or shape retention are critical; however, the presence of Nickel in the alloy raises concerns about allergic and immunological reactions. This makes NiTi a good testing bed for corrosion related studies. Controlling the corrosion mechanisms of NiTi alloys is crucial step for their wider use in medical field as the risk of uncontrolled Ni ion leaching from the implant surfaces is an ongoing concern. Aksakal et al. have analyzed the influence of released ions from common implant materials in order to characterize their potential effect on the human body that summarized in Table 1 (Aksakal et al., 2004).

**TABLE 1:** Potential adverse reactions caused by releasing of metal ions [for more information on immune reactions, please see (Kämmerling, et al., 2021)].

Materials (ionic and/or inorganic states)	Effect of leaching to human body
Nickel	Affect skin, pneumonia, chronic sinusitis
Cobalt	Anemia B
Chromium	Ulcers and central nervous system disturbances
Aluminum	Epileptic effects and Alzheimer’s disease
Vanadium	Toxicity via induction of excessive reactive oxygen species production

Corrosion resistance is one of the first and foremost required properties for all implantable materials and the corrosion have been seen as a problem for thousands of years, long before any effective preventive methods were discovered. Researchers focus on controlling the possible reactions between living tissues and implant material surfaces in order to prevent harmful effects for enhancing the life span of the implants, reducing the possibility of revision surgeries due to medical complications (aseptic loosening, infection, mechanical failure). It is known that all implantable metal-based materials face an aggressive, corrosive environment due to the highly corrosive nature of blood and other constituents of the body fluids that may trigger the corrosion mechanisms of metal-based implant materials (Manivasagam et al., 2010).

Corrosion stands for a long-term, natural process caused by chemical and/or electrochemical reaction of metals with the environment. Gas or liquid in contact with metal will initiate oxidation, due to the metal’s tendency to reach chemically more stable state. Resistance to corrosion differs from metal to metal. Some metals have high resistance to corrosion, like gold and platinum, which can be found at their pure state in nature (Eliaz 2019). Some of the metals, like zinc, can be considered as highly resistant to corrosion due to slow kinetics of the process, meaning that corrosion happens but very slowly. Aluminum, stainless steel, and titanium are widely used due to high corrosion resistance (Ahmad 2006), which comes from passivation. Passivation refers to spontaneous formation of a very thin layer at the metal surface, consisting of corrosion products, that serves as a barrier and protects the metal from further corrosion. In the context of implantable materials, the consequences are generally more substantial as the loss of function of the implant due to corrosion will require an additional surgery and the release of corrosion products will have biological effects both in the vicinity of the implant and at systemic level.

The corrosion process itself is a very complex process which includes several phenomena and is influenced by many factors. In general, the corrosion damage should include not only physicochemical and environmental parameters, but also different parameters of a stochastic nature (Pidaparti et al., 2008).

In order to be safe for usage and to have a good biocompatibility, various tests need to be performed on biomaterials before any clinical trial and application. Although some expected situations can be used to set up experiments, it is hard to experimentally predict complete behavior of the biomaterial, in terms of corrosion process due to its stochastic nature. This is particularly relevant for the testing of the effects of the corrosion products on cells *in-vitro*; as the *in-vitro* cell culture timescales are not in line with the corrosion timescales. The second problem with corrosion experiments is that they last long and collecting experimental data is time consuming. These are the reasons why computational modelling is required and desirable for analysis of corrosion behavior. Computational/*in-silico* models reduce greatly time of collecting data and allow us to include different parameters in calculation. Therefore, *in-silico* modelling of corrosion should integrate various influencing parameters from solid mechanics, surface- and electrochemistry, materials science, probability and statistics, and fracture mechanics (Wei and Harlow 2003). Corrosion quantification should also include not only loss of thickness, but also morphology of the corroded area. The results obtained in literature indicate that classification of corrosion pits is possible with image analysis and may be used for correlating service/failure conditions based on corrosion morphology (Choi and Kim, 2005).

Computational modelling of corrosion process can be conducted at different levels–from macro to micro level. At the macroscopic scale, modelling of corrosion is focused on solving differential equations with numerical methods such as finite element method (FEM), FDM (finite difference methods), BEM (boundary element methods) etc. (Gunasegaram et al., 2014; Macdonald 2011; Thébault, et al., 2012), although these are basically deterministic (Féron and Macdonald 2006). The mathematical models of electrochemical reactions in corrosion are usually regulated using the reaction-diffusion equations. This kind of model has been widely discussed in a number of publications (Fonna et al., 2013; Fonna et al., 2016). BEM is a common choice in the field of numerical methods used for modelling of corrosion; however, it can only be used to measure the corrosion at the surface. Since corrosion is primarily a surface process, this approach saves a lot of computational time. Previous studies on pitting corrosion with stochastic method are available, including the probabilistic models (Yuan et al., 2009), the Markov-based model (Valor, et al., 2013) and the Monte Carlo approach (Caleyo et al., 2009). These methods do not include the electrochemical basis of corrosion, which has been introduced with the stochastic model called Cellular Automata (CA). CA is a tool commonly used for modeling non-linear mechanisms such as diffusion of reactions (Schiff 2011), bio-morphogenesis (Ermentrout and Edelstein-Keshet 1993), inflammatory response of immune cells (Ibrahim and Pidaparti 2017). On a smaller scale, these phenomena usually display stochastic impressions, but as the scale grows, they tend to show clear trends. CA is commonly used to model these processes, because of its practicality in execution. Besides them, there are also some CA versions available for modelling aircraft pitting corrosion.

The original CA model suggested by Von Neumann is a two-dimensional square bar, in which each square is called a cell. At any given moment, each of these cells may be in a different state. The evolution of each cell and the modification of their internal states proceed synchronously and are regulated by a series of laws. Thus, generated cellular space is a complete discrete dynamic structure. Earlier studies show that CA as a discrete dynamic system displays many of the features of a continuous dynamic system, but in return CA offers a simplified structure. This particular feature of CA makes it the ideal computational approach to corrosion model development (Aparti et al., 2005). Pidaparti et al. developed a CA that modeled the rate of pit spread once initiated, with later addition of interaction between pits in multi-pit growth (Pidaparti, M; Fang, L and Palakal, J 2008). Another model by the same group of authors referred to pitting corrosion as a mixed system that included coupled deterministic-probabilistic simulations of pit growth (Ibrahim et al., 2018). These models were mostly two-dimensional, and did not include the growth in depth (third dimension). Li et al. (Li, et al., 2009) and Di Caprio et al. (Di Caprio, et al., 2011) proposed two-and three-dimensional pit cavity growth models. Their models were based on full CA stochastics laws. Sometimes, described models are coupled with artificial intelligence methods to further predict corrosion. Specifically, CA based simulation of corrosion pit initiation and growth, wavelet-based imaging methods for corrosion risk estimation, and artificial neural networks (ANNs) for material failure and residual strength predictions were described in (Pidaparti 2007). While ANNs do not include any empiric, deterministic or physical features of the localized corrosion mechanism, they can be used to forecast future progress as a function of different parameters (Pidaparti et al., 2005).

In order to characterize corrosion damage growth qualitatively and quantitatively, image analysis had to be performed. Some papers discussed the concept of creating textural/color features that are resilient to corrosion images, using a low-tech method that uses a commercial color scanner (Pidaparti, Hinderliter and Maskey 2013; Medeiros, et al., 2010). An investigation of the textural characteristics of wavelet transformations and color features was performed to define the corrosion damage metrics during corrosion growth under three different electrolyte solutions (Pidaparti et al., 2013). In order to explain corrosion development in time (Pidaparti et al., 2013), the strategy by Pidaparti et al. was to combine functionality mitigation properties due to material compromise (textural features) with local aspects (color values). Kapsalas et al. (Kapsalas et al., 2007) suggested a system for detecting corrosion size and topology in stonework surfaces by checking and analyzing image segmentation schemes. They also demonstrated that their analyses were in strong alignment with assessments focused on chemical analyzes carried out on the same surfaces. Choi et al. (Choi and Kim 2005) analyzed surface corrosion damage using optical image processing techniques. Model interpretation was based on co-occurrence matrix, and multidimensional scaling method, used to define images by three types of color, texture, and form elements. Wang et al. (Wang and Song 2004) used wavelet packet decomposition energy of images in various wavelet sub-bands as a feature to analyze the atmospheric corrosion activity of zinc samples. They also acquired a relationship between the chosen image features and the corrosion weight loss. Tao et al. (Tao, et al., 2008) analyzed atmospheric corrosion of field exposure to high strength aluminum alloys. They used wavelet-packed decomposition energies with various sub-bands to measure the corrosion loss of five types of aluminum alloys. On the basis of wavelet transforms and fractals, the corrosion morphology of nickel-aluminum-bronze metal was analyzed under various corrosion and stress conditions by Pidaparti (Pidaparti, et al., 2010). Another work by the same author, suggested the usage of histogram features including mean, standard deviation, skew, energy, and entropy for the analysis of corrosion images (R. Pidaparti 2007). In the context of biomaterials, particularly the use of metals, the image acquisition method of choice is Light and Scanning Electron Microscopy. This is primarily due to the fact that the microscopic features of the implant surfaces, even the nanoscale features such as nanotubes, have a direct effect on the functionality of the implant. Thus, an *in-silico* model of corrosion in the biomaterial context should be able to use such images.

In addition to numerous deterministic and empiric approaches that can be found the literature, there is still a need to develop numerical models that can predict the corrosion growth morphology, as well as influence of variety of environmental parameters. The literature review showed us that there is no simulation of the corrosion that includes all of the phenomena. We consider that it is important to put a focus on an important correlation between microscopic and macroscopic approaches, which can be achieved using CA. A significant contribution of this type of method represents the integration of chemical and electrochemical aspects in the evolution of morphological aspects (Zenkri, et al., 2017). This will also enable to predict the outcome of the corrosion process and link it with *in-vitro* tests. This paper first introduces the experimental procedure for corrosion tracking, then implements the *in-silico* simulations of corrosion initialization and progress, followed by the investigation of the influence of different environmental parameters, using experimental results from a NiTi alloy as an example.

## Materials and Methods

### Experiments

Over the past decade, many aspects of corrosion mechanism have been studied and remained controversial in order to offer detailed understanding on *in vivo* and *in vitro* studies. Generally, the corrosion behavior of metallic materials has been investigated in body-like mediums including phosphate buffer solution (PBS), simulated body solutions, Hank’s solution, NaCl solution, Ringer solution and artificial saliva at specific pH values in order to mimic the human body condition.

The potentiodynamic tests were utilized in order to test the corrosion resistance of NiTi shape memory alloys at different parameters. Prior to testing, the cleaning process of NiTi samples was carried out using the ultrasonic bath with acetone, ethanol and deionized water, respectively. Electrochemical measurements were obtained utilizing a Gamry Potentiostat/Galvanostat (model 1,000 Interface) to determine the corrosion resistance of the samples in a simulated body fluid (SBF) to simulate the body environment. During the experiment, NiTi samples were placed in a Teflon sample holder and exposed to the electrolyte solution. A conventional three-electrode electrochemical cell was used. NiTi wires were used as the working electrode while a platinum wire was used as the counter electrode and a saturated calomel electrode was used as the reference electrode. Simulated Body Fluid was utilized as the electrolyte with pH 7.4 at body temperature. Potentiodynamic scans were performed with scan range from −0.6 to 0.3 V with a scanning speed of 10 mV/s. SBF solution was prepared according to a procedure described by Kakubo (Kokubo and Takadama, 2006). All reagents were dissolved one by one as given in Table 2 in order to prepare 1 L solution.

**TABLE 2:** Reagents for SBF.

Reagent	Amount/l
NaCl	7.996 g
NaHCO_3_	0.350 g
KCl	0.224 g
K_2_HPO_4_.3H_2_O	0.228 g
MgCl_2_.6H_2_O	0.305 g
1M HCl	40 ml
CaCl_2_	0.278 g
Na_2_SO_4_	0.071 g
(CH_2_OH)_3_CNH_2_	6.057 g

Electrochemical corrosion measurements were carried out at body temperature, 37°C. First test was to measure the open corrosion potential for 10 min, followed by a potentiodynamic experiment. During the experiments, a conventional three-electrode electrochemical cell was used. NiTi samples were placed in a Teflon sample holder on which a 10 × 10 mm area was exposed to the electrolyte solution. SBF was used as the electrolyte in 100 ml solution volume. In order to take the steps towards the validation of the corrosion modelling studies, different parameters were obtained in corrosion experiments. One set of tests were utilized with pH at 7.4 and 9 and the other sets were obtained at room temperature and body temperature. A number of publications proposing different types of environments can be found in the literature while a couple of studies have been published by Figueira et al. (2009) that relies on the effect of pH. In their study, the range of selected pH values were between pH 3 to 10 to simulate the condition that could be resulted in possible inflammatory and allergenic reaction in the human body since pH is one of the most important parameters that alter the corrosion mechanisms. In the present study, we aim to investigate the corrosion behavior of NiTi materials by using potentiodynamic polarization method for different environmental conditions. All experimental efforts were conducted in simulated body fluid (SBF) with pH 7.4 and 9 while other modeling parameters were selected in order to have comprehensive investigation. The range between pH 7 to 10 was utilized to simulate the case of prolonging duration of stay under the harsh conditions in particular for long term implantation since this case may be resulting in the ion release from the implant surface.

Optical microscope analyses were utilized for providing the observational image-based understanding for developing simulation model. A microscope attached to the camera was employed at different magnifications of 20x and 50x for all samples.

### *In-Silico* Modelling of the Corrosion

**FIGURE 1:**
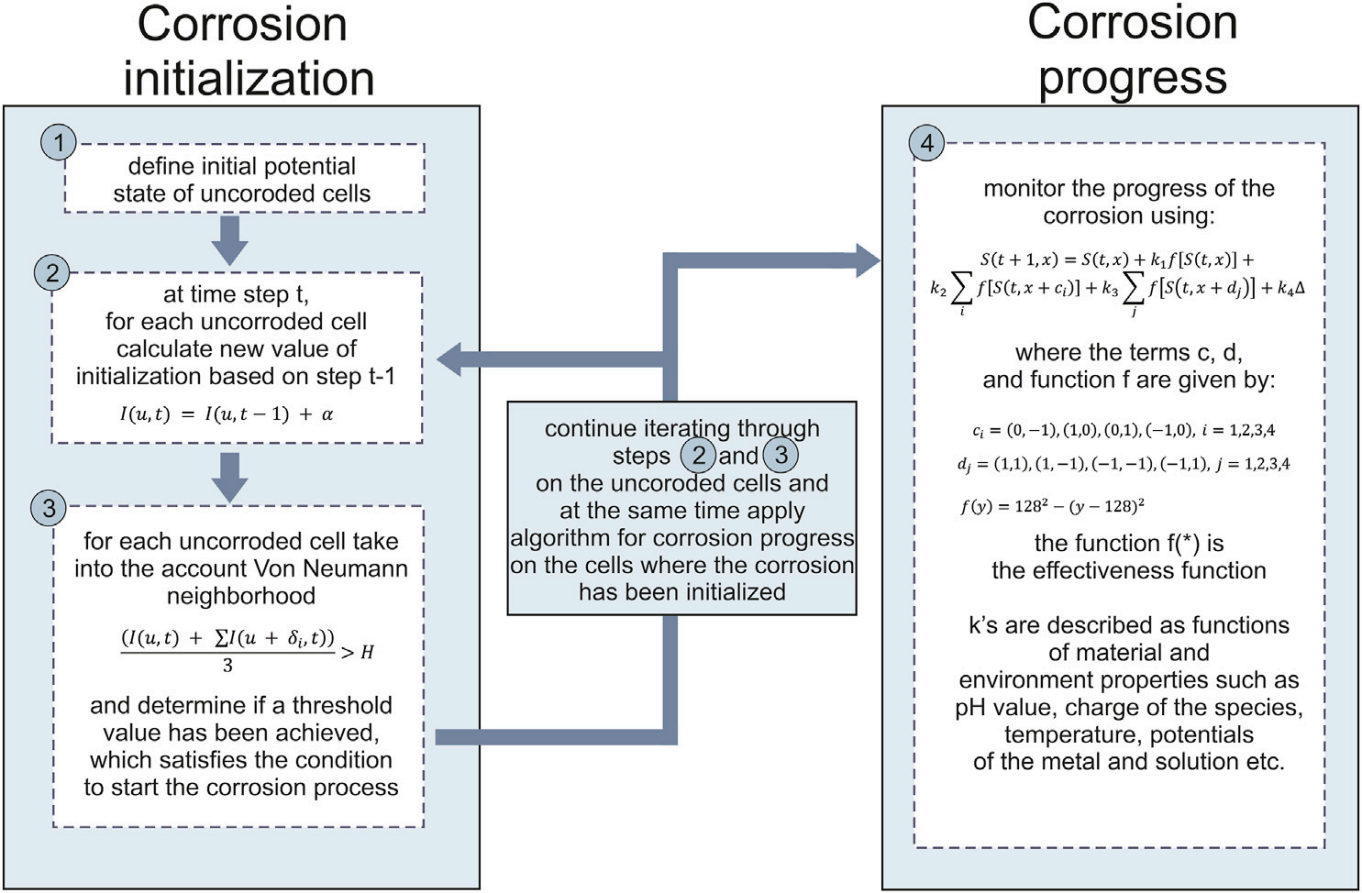
Flowchart of the corrosion modelling.

*In-silico* corrosion model was developed on the basis of the Cellular Automata. The evolution of each cell in CA happens through a sequence of synchronous updates of all cells, which are regulated by a set of functions (rules). The computational modelling of multi-pit corrosion in medical implants based on cellular automata is divided into two sub-models–corrosion initialization and corrosion progress models. The state of each cell has been represented by a predefined interval in the range of 0–255, where uncorroded cell has the value of 0 and totally corroded cell the value of 255. This means that the corrosion result is represented with an image of the material surface, where certain rules were prescribed to follow the cells where the corrosion has been initialized and will progress in time (Figure 1). The image used to model the corrosion of the material was 200 × 200 pixels, while the user can change the number of time steps (number of time steps is the input parameter of the simulation).

**FIGURE 2:**
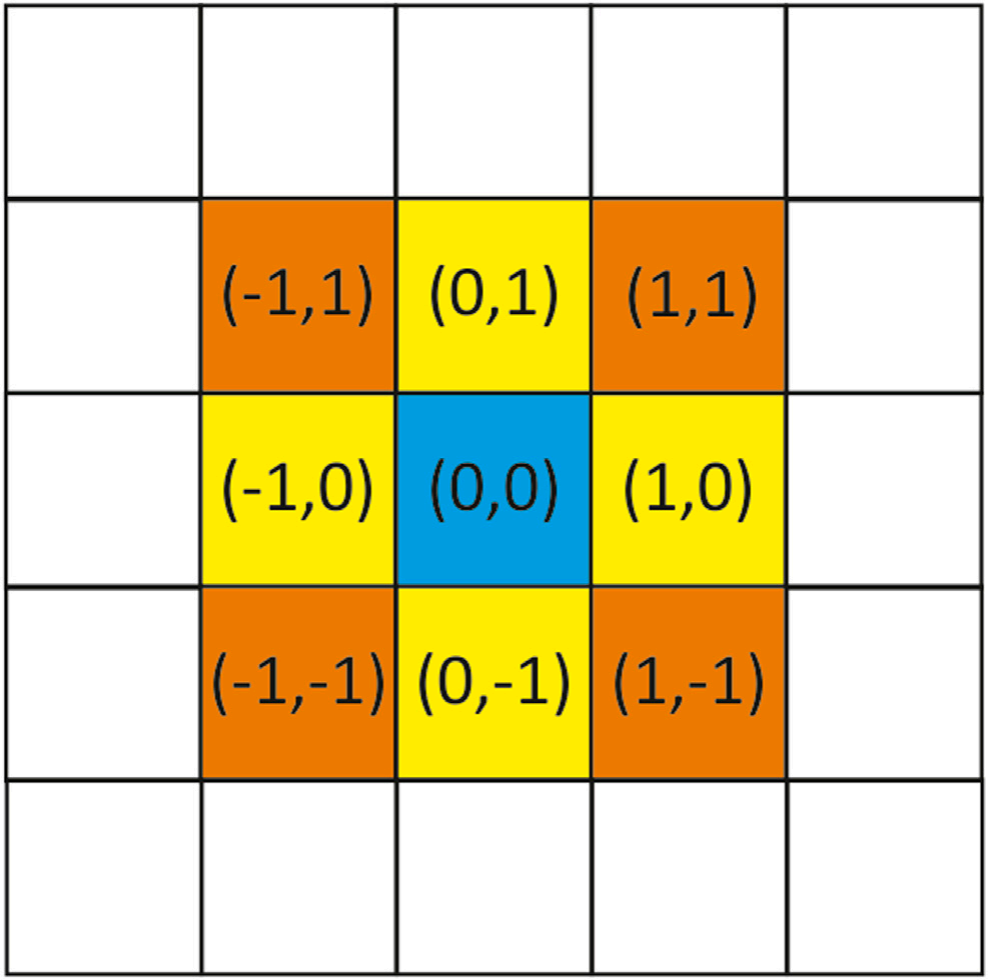
Area of image denoting Moore’s neighborhood.

As mentioned above, we represent a material of interest as two-dimensional square lattice in which each square is called a cell. In our case each cell would be a pixel in image. Each of these cells can be in a different state (different pixel value) at any given time. The evolution of each cell and the updating of the internal states of each cell occur synchronously and governed by a defined set of equations. At the beginning of the simulation (t=0), we assume there is no corrosion of the material, meaning the first step is the black image, meaning all the states of the pixels are 0. After that, corrosion initialization has been started, each cell x is associated with an initial potential state I(u,t):

I(u,t)=I(u,t−1)+α (1)

where α is an increment of the pixel value (state potential). For each uncorroded cell u, we set initialization potential state according to the Von Neumann neighborhood algorithm in the form $I\left(u,t\right)+\;\sum I\left(u+\delta_{i},t\right),\;i=1,2,3,4$. The parameter $\delta_{i}$ represents the Von Neumann neighborhood (up, down, left, right). If that sum divided by 3 becomes larger than a certain threshold, then a corrosion has been initialized at cell u and the corrosion state S(u,t) (pixel value) of that cell is set to a small positive number. For the following time steps, corrosion initialization sub-model is applied on all the other uncorroded cells again, and at the same time corrosion progress sub-model is applied on the cells where corrosion has been initialized using the equation:

$$S\left(t+1,x\right)=S\left(t,x\right)+k_{1}f\left[S\left(t,x\right)\right]+k_{2}\sum_{i}f\left[S\left(t,x+c_{i}\right)\right]+k_{3}\sum_{j}\left[S\left(t,x+d_{j}\right)\right]+k_{4}\Delta$$ (2)

where $c_{i}=\left(0,-1\right),\left(1,0\right),\left(0,1\right),\left(-1,0\right)$, $d_{j}=\left(1,1\right),\left(1,-1\right),\left(-1,-1\right),\left(-1,1\right)$ for i=1,2,3,4 and j=1,2,3,4. This means that we implemented the Moore neighborhood to describe the influence of the surrounding cells to the cell of interest (Figure 2). In this case $c_{i}$ represent the cells down, up, right, and left from the cell of interest, while $d_{j}$ represents the cells diagonally up-right, up-left, down-left, and down-right from the cell of interest (Figure 2).

Function f is in the form $f\left(x\right)=128^{2}-{\left(x-128\right)}^{2}$ or after rearrangement $f\left(x\right)=\;-x^{2}+256\cdot x$. This shows that the effectiveness functions are not uniform, but should be the same for symmetric neighbors. The shape of function f(x) is a parabola, due to the fact that the cell changes from uncorroded to partially corroded and finally to fully corroded (0–255), while the activity of the chemical reaction increases from zero up to some point then falls back down to zero. In order to account for stochastic effects in corrosion, Δ factor is added as a standard random variable with mean 0 and variance 1.

Based on the review of the literature (Macdonald and Urquidi-Macdonald 1992; Pidaparti et al., 2008), coefficients k can be expressed in terms of the chemical parameters that affect the corrosion growth. We adopt the parameters influencing corrosion suggested by (Pidaparti et al., 2004) and derived based on experiments from (Macdonald and Urquidi-Macdonald 1992). Coefficient $k_{1}$ is described as part of the continuous mathematical model used to find out which parameters affect the pitting process and how they affect the growth rate:

$$k_{1}=\lambda \cdot {\left(pH-7\right)}^{2}\cdot step\left(4,8.5\right)\cdot e^{\varphi_{M}-\varphi_{S}}\cdot \left(1/T\right)\cdot C\cdot D\cdot z$$ (3)

where λ is a discount factor that ranges from 1 to 3; pH is the pH value of the solution; *step (4, 8.5)* is a function with value 0 in the range of 4 and 8.5, and 1 otherwise; $\varphi_{M}$ and $\varphi_{S}$ are the potentials of the metal and solution, respectively; T is the absolute temperature; C is the concentration of the reaction species; D is the diffusivity of the reaction species; z is the charge of the reaction species. The form of parameters $k_{2}$, $k_{3}$, and $k_{4}$ is similar to the $k_{1}$, differencing only in a discount factor. Discount factor has the meaning of the influence of the neighboring cells - the farther the cells are from the cell of interest, the less effective role the neighbors take in the corrosion of that cell and therefore discount factor is smaller.

Pseudocode for the program is as following:


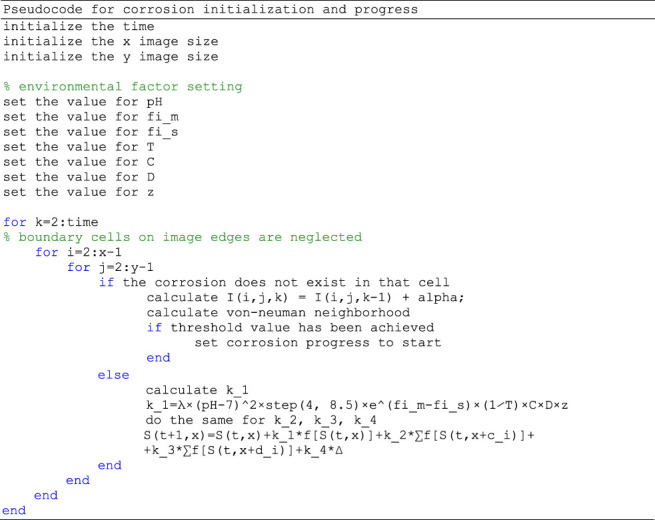


*In-silico* model is implemented in Matlab 2017a, due to the fact that matrices and arrays are the fundamental representation of information and data in Matlab. Although the proposed model by definition uses sequential updating of the rules for each cell (pixel), Cellular Automata models are suitable to be effectively and naturally implemented on parallel computers achieving high performance. The model was developed and tested on a computer with processing hardware of 8GB of RAM, a GPU Nvidia GeForce GTX960M, and an Intel (R) Core (TM) i7-6700HQ CPU at 2.60 GHz. At this point, running our model and obtaining the results on such computer lasts for less than 1 min, therefore parallelization is not necessary at the moment, but can be achieved in the future.

As can be seen we take into account different environmental factors in order to describe their effect on the corrosion. We will further analyze the influence of different environmental factors on corrosion. These factors, which represent the inputs to the simulation of the corrosion with corresponding ranges are given in Table 3.

**TABLE 3:** Inputs for the corrosion model.

Name of the parameter	Label	Explanation	Unit	Range	Default value
pH value	pH	pH value of the solution	—	7–10	7.4
Potential of the metal	$\varphi_{M}$	change in a corrosion system of the metal	V	0.1–1	0.23
Potential of the solution	$\varphi_{S}$	change in a corrosion system of the solution	V	0.1–1	0.2
Absolute temperature	T	Absolute temperature of the environment	K	297.15–313.15 (24–40°C)	310.15
Concentration of the reaction species	C	Concentration of one of the species participating in a corrosion reaction	M/dm^3^	0.1–0.5	0.2
Diffusivity of the reaction species	D	The rate of diffusion-controlled corrosion of reaction species	dm^2^/s	0.1–0.5	0.3
Charge of the reaction species	z	Charge resulting from the reaction of species	Faraday	0.1–0.5	0.2
Time of simulation	t	Number of time steps to run the simulation (number must be divisible by 20, as every 20 steps are plotted)	—	1,000	100

It should be noted that proposed ranges ensure the stability of the model and are derived based on review of the literature. The series of values used in these papers are derived from experimental data obtained from Center for Materials Diagnostics at the University of Dayton Research Institute (Pidaparti et al., 2005). However, these ranges are only proposed for the platform integration and model itself is not limited to testing with only these ranges.

In order to relate physical time versus time step, a methodology presented in (Di Caprio et al., 2016) will be employed. That means that space and time equivalence for our pixel size and simulation time step need to be defined in terms of real dimensions. In this study, surface of 0.2 × 0.2 mm relates to 200 × 200 pixels image, while the time scale for the investigated experimental corrosion was 4 days, upon which the whole area was corroded, meaning that the corrosion rate determined in experiment was approximately 20 mm/year. Taking into account neutral environment conditions, pH = 7 at room temperature case for NiTi, the corrosion rate is 774 mil/year (1 mils = 0.0254 mm). If we consider that 200 × 200 pixels image stands for 0.2 × 0.2 mm experimental sample, the length of one pixel is 0.001 mm = 1 µm (physical area size of one pixel is 1 µm x 1 µm). The corrosion in experiment is complete in 4 days and with simulation in 100 time steps (in the figures time = 20 time steps) so in that case one time step is 57.6 min. Taking into account obtained value of one time step, the values presented in the figures–time = 1, 2, 3, 4, and 5 corresponds to 19.2, 38.4, 57.6, 76.8, and 96 h, respectively. The same methodology can be applied for any type of surface and material in order to establish the connection between the physical time and time step, which simplifies further discussion.

### Image Analysis of the Corrosion Process

Output of the simulation of the corrosion model are given in the form of images for every time step. In order to quantitatively describe the outputs, the second part of the proposed methodology includes the analysis of *in-silico* obtained corrosion images. Besides that, the user can upload experimentally obtained corrosion images to our web platform, described in Supplementary Materials. After this, the calculation software can be used to compare the results obtained experimentally and from simulations.

Upon finishing the simulation, we use resulting images to quantitatively estimate the corrosion progression over time by calculating the following statistical measures (Table 4). These measures are calculated using image processing techniques and are all based on pixel intensities and image histogram probabilities. More detailed explanation of how each measure is calculated in image processing is given in Table 4.

**TABLE 4:** Investigated statistical measure for estimation of corrosion progress.

Name of the measure	Explanation
Mean corrosion	average grey value of the grayscale’s image histogram probability
Standard deviation	describes the spread of the data set and is related to image contrast
Skew	asymmetry about the mean value in the distribution
Percentage of corroded material	sum of the corroded pixels (all non-zero values) divided by number of pixels and multiplied by 100
Kurtosis	determines whether the data are peaked or flat relative to a normal distribution
Energy	indicates how the pixel intensities are distributed in the region under consideration. In the case of corrosion image, this feature indicates the degree of corrosion at the pit level
Entropy	indicates the number of bits we need to code the image data
Power	indicates the texture property in an image and in this case, the level of corrosion itself. The higher the power value, the texture change will be higher as well
Contrast	difference between maximum and minimum pixel intensity in an image
Wavelet features	calculated through the processes of singular values decomposition (SVD); only the first two eigenvalues are reported

The importance of these measures lies in the fact that image analysis has been used to characterize corrosion morphology in materials subjected to a variety of environmental conditions (Choi and Kim 2005). For this purpose, we have used several image analysis methods for characterizing the corrosion surface morphology–primarily wavelet transformation and then energy, entropy calculations. In order to bring the research closer to the end-users, a user-friendly web platform that follows the workflow of two main sections (corrosion model and corrosion experiment) was added to this study. This is given in Supplementary Materials. Sequence diagrams (Supplementary Figure S1), as well as User interface for in silico simulations (Supplementary Figure S2) and analysis of Experimental images (Supplementary Figure S3) are given. Related to discussion of results, Supplementary Figure S4, S5 discuss the format of obtained results for the user.

## Results and Discussion

### Simulation Results

**FIGURE 3:**
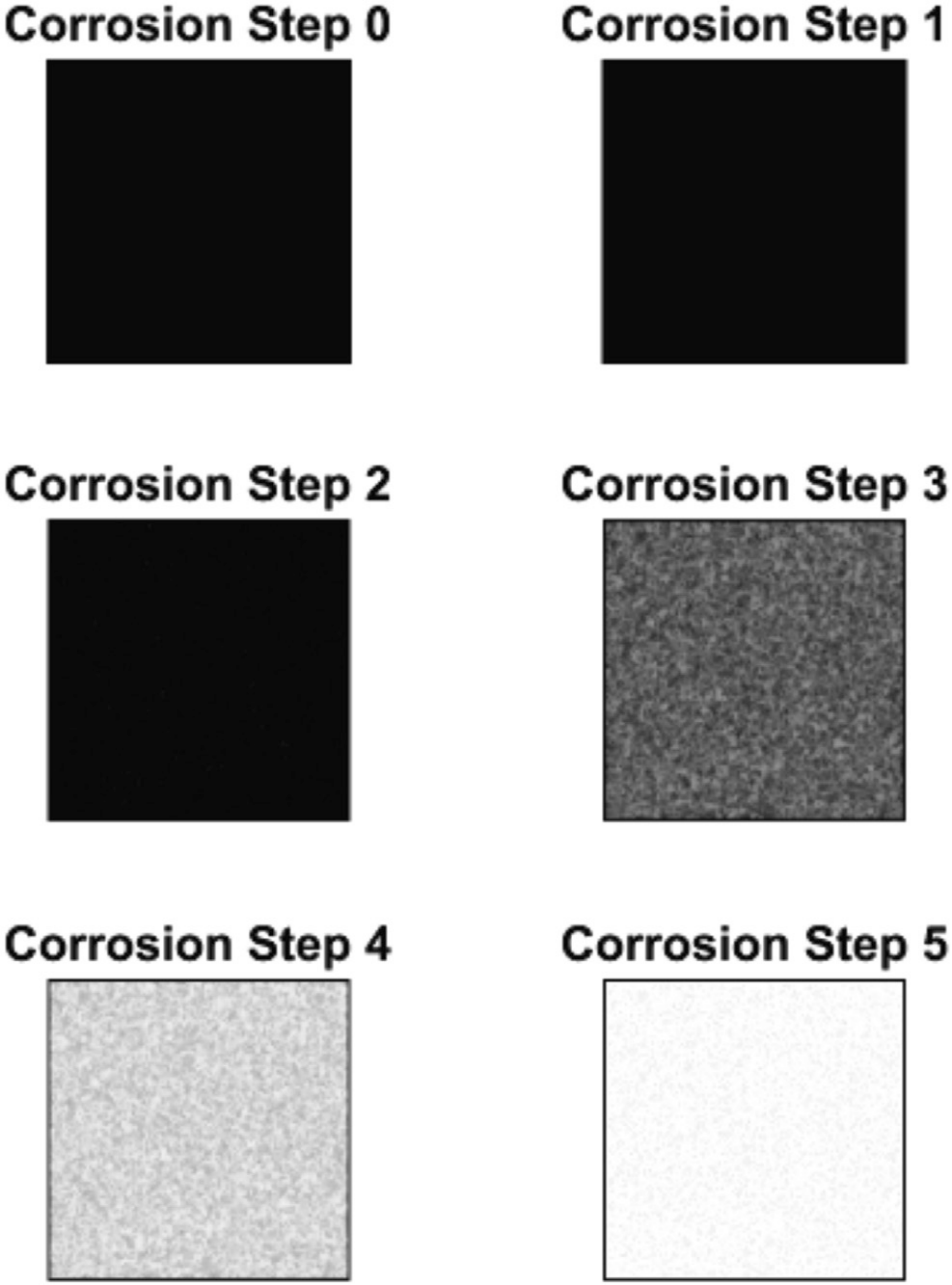
Corrosion state S(u,t) at time steps t=0,1,2,3,4,5. Taking into account the experimental work in this paper, the time steps 1,2,3,4 and 5 correspond to 19.2, 38.4, 57.6, 76.8 and 96 h, respectively.

The results of the corrosion simulation are displayed in the form of images and analysis of the resulted images (described statistical measures) that describe the corrosion in time. Figure 3 shows the corrosion states at six different time steps. Black pixels indicate uncorroded cells and white indicate fully corroded cells. Adopted values for parameters used in this simulation are pH=7.4; $\varphi_{M}=0.23\;V$, $\varphi_{S}=0.2\;V$, T=310.15 K, C=0.2, $C=0.2M/dm^{3}$, $D=0.3m^{2}/s$, z=0.2 Faraday.

In Table 5, we present the example of numerical results from corrosion simulation model. Zero state is always the starting point (no corrosion has been initialized; therefore, all the values are initialized–zero, NaN or 1). In each time step, values for all statistical metrics are calculated. It should be emphasized that platform displays images and calculates statistical measures for every t=20∗n time steps to ensure the corrosion progress is visible.

**TABLE 5:** Example of results based on analysis of images.

	Steps					
	0	n = 1	n = 2	n = 3	n = 4	n = 5
Mean	0	0	2.397425	3.473625	5.044675	6.902025
Standard deviation	0	0	2.379,689	2.968312	3.784652	5.154516
Skew	NaN	NaN	0.071398	−0.21147	−0.45735	−0.47701
Corroded area (%)	0	0	51.4875	59.2	65.48	65.48
Kurtosis	NaN	NaN	1.173598	1.2381	1.454129	1.468136
Energy	1	1	0.339813	0.274002	0.222435	0.202722
Entropy	0	0	1.772208	2.099116	2.420434	2.635617
Power	0	0	1.83E+10	3.34E+10	6.36E+10	1.19E+11
Contrast	0	0	0	0	0	0
wavelet features (S1,S2)	0	0	1.899763	2.755,384	4.005375	5.483206
	0	0	0.394938	0.501356	0.622364	0.802802

In such a way, several metrics can be tracked and results from simulation and experiments can be compared. Besides that, the influence of each parameter in the simulation can be investigated in order to optimize the conditions and extend the time for corrosion (prolong the process of corrosion).

In that sense we have performed a thorough investigation of the influence of all the environmental factors included in the model. Here we present mainly the results related to mean corrosion parameter and entropy when variations of different environmental factors–pH, potential difference, temperature, concentration of the reaction species, diffusivity of the reaction species and charge of the reaction species. Of the various features considered, in literature it was found that entropy showed significance with various parameters in the corrosion damage process (Pidaparti et al., 2005). It was shown that entropy can be used as an indicator of the corrosion material loss. Pidaparti et al. claim that the energy and entropy features correlate well with experimental data as compared to other statistical features (Pidaparti et al., 2005). From the definitions of entropy, it can be concluded that the wavelet entropy is minimum when the image represents an ordered activity characterized by a narrow frequency distribution, whereas the entropy is high when an image contains a broad spectrum of frequency distribution.

**FIGURE 4:**
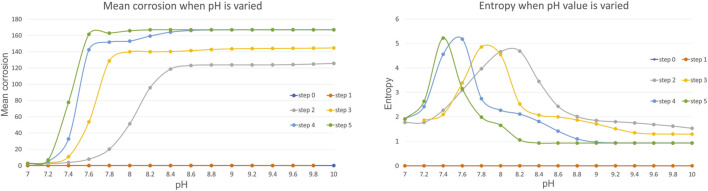
Results for mean corrosion **(left)** and entropy **(right)** when pH value is varied.

Firstly, we have investigated the influence of pH value which is varied in the range of 7.4–10, with an increment of 0.2. From all the calculated metrics we have found mean corrosion and entropy to be the most illustrative to discuss. Figure 4 shows these metrics for the variation of pH.

The results show that the increase in pH value in the range of 7–10, increases the slope of faster corrosion, and for example for the step 5, high mean corrosion has already been achieved with pH = 7.6. The greater the pH value, the greater the mean corrosion is, also confirmed by experiments in this study presented in *Experimental Results* (Table 6). As expected, the greater corrosion is achieved at later steps. The results for entropy are very interesting, since after certain threshold, the entropy reduces and converges after the value of pH = 8.8–9, which also corresponds to the fixed value of mean corrosion. The results are also in accordance with (Capoşi et al., 2011) that showed that with the increase of pH value, the corrosion increases, after which in becomes stable.

**TABLE 6:** Potentiodynamic polarization results of samples for pH conditions.

pH value	Corrosion rate (mpy)	Error
7.4	774.2	0.4
9.0	947.8	0.2

**FIGURE 5:**
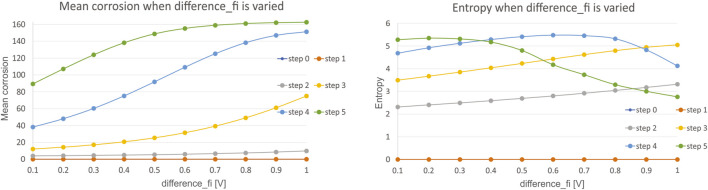
Results for mean corrosion **(left)** and entropy **(right)** when $\varphi_{M}-\varphi_{s}$ is varied.

Secondly, we have investigated the influence of potential difference $\varphi_{M}-\varphi_{s}$ which was varied in the range of 0.1–1 V, with a step of 0.1 V. It was not necessary to investigate a separate influence of each parameter, as only the potential difference $\varphi_{M}-\varphi_{s}$ is present in the governing equations for corrosion. Mean corrosion and entropy for the variation of this parameter is presented in Figure 5.

The results show that with the increase in difference $\varphi_{M}-\varphi_{s}$ in the range of 0.1–1, we can notice slow increases of the mean corrosion, which is in accordance with (Trépanier and Pelton 2006) and (Ahmad 2006). The explanation for such trend change is that higher potential difference increases both the chemical reaction rate and the mass transport rate, which in turn increases the corrosion rate. The slopes are less steep than during the variation of pH value. The results for entropy show the same conclusion, as the values for entropy are tending to be constant, indicating that this range difference $\varphi_{M}-\varphi_{s}$ does not have as much influence on the corrosion as pH value does.

**FIGURE 6:**
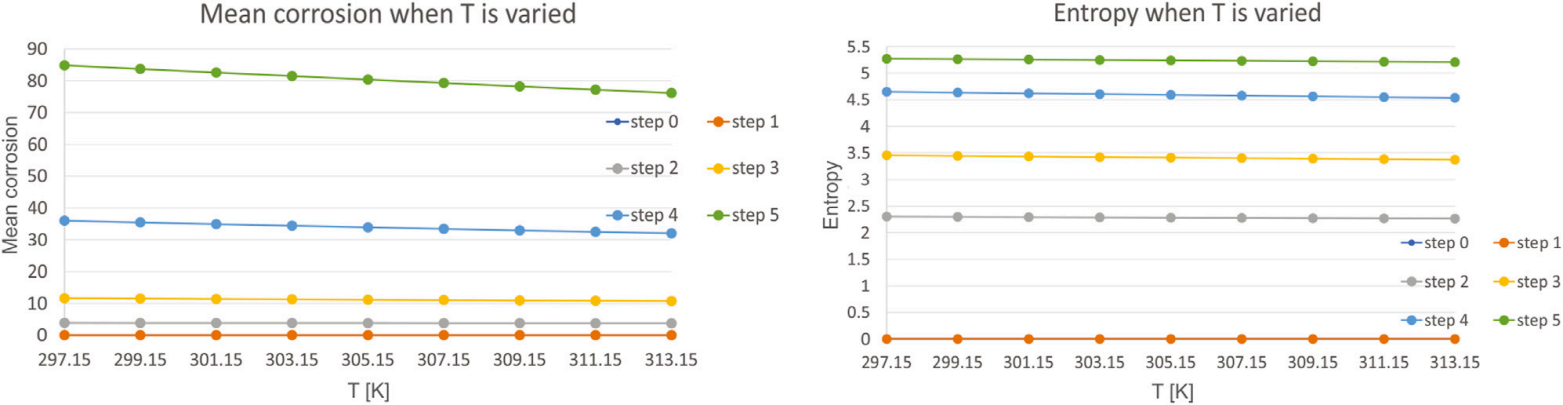
Results for mean corrosion **(left)** and entropy **(right)** when temperature T is varied.

Thirdly, we have investigated the influence of temperature T, which is varied in the range of 297.15–313.15 K, with a step of 2 K. Mean corrosion and entropy for the variation of this parameter is presented in Figure 6.

The results are even more interesting for temperature, as it was shown that the investigated range of 297.15–313.15 K, which is equivalent of 24–40°C do not influence the corrosion almost at all. As the temperature increases, for each step, there is even also a slight decrease in highest value of mean corrosion. Investigation of the entropy leads to the same conclusion. These results are in line with the experimental studies from literature, such as (Ahmad 2006). Their studies have shown that the effect of temperature on corrosion rate is minimal at low temperatures i.e. body to room temperature. Itn literature it was also shown that there is no major effect on corrosion in the investigated ranges (Trépanier and Pelton 2006). The same conclusions are met in the 3.2. experimental section of this study.

**FIGURE 7:**
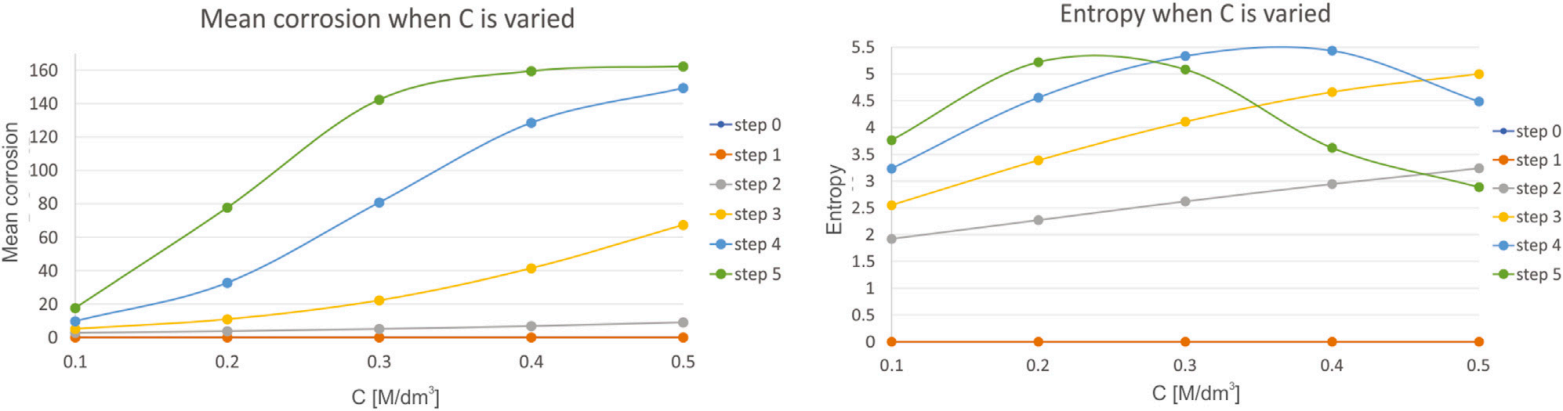
Results for mean corrosion **(left)** and entropy **(right)** when concentration of the reaction species C is varied.

Fourth, we have investigated the influence of concentration of the reaction species C, which is varied in the range of 0.1–0.5, with a step of 0.1. Mean corrosion and entropy for the variation of this parameter is presented in Figure 7.

The influence of concentration of the reaction species C are similar to the results performed during investigation of the influence of pH value, but with a higher impact than pH had. As an example, consider that the mean corrosion converges towards maximal value of 160 M/dm^−3^ even for the smaller ranges (0.1–0.5) of this parameter, compared to same range change of pH value (7–7.5) when maximal mean corrosion achieves value of only 80. Similar can be concluded when analyzing entropy. A decrease in entropy is achieved faster with variation of C, than with pH, considering the same ranges of change of their units. Again, the same conclusion is met in (Trépanier and Pelton 2006), since with the increase in concentration species, both the mass transport rate and the chemical reaction rate are going to be influenced.

**FIGURE 8:**
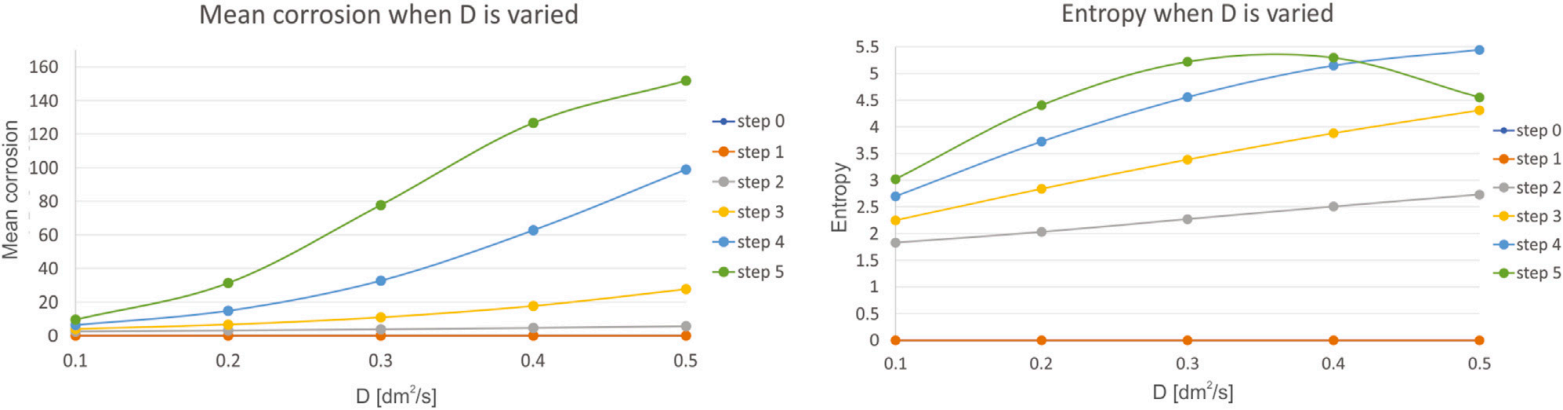
Results for mean corrosion **(left)** and entropy **(right)** when diffusivity of the reaction species D is varied.

Fifth, we have investigated the influence of diffusivity of the reaction species D, which is varied in the range of 0.1–0.5, with a step of 0.1. Mean corrosion and entropy for the variation of this parameter is presented in Figure 8.

The diffusivity of the reaction species D tends to have the effect similar as the reaction species C, however with a smaller impact, as slopes are not steep and maximal values are less during variation of this parameter. The trend is similar compared to the variation of the reaction species C, but the effects on level of corrosion are smaller.

**FIGURE 9:**
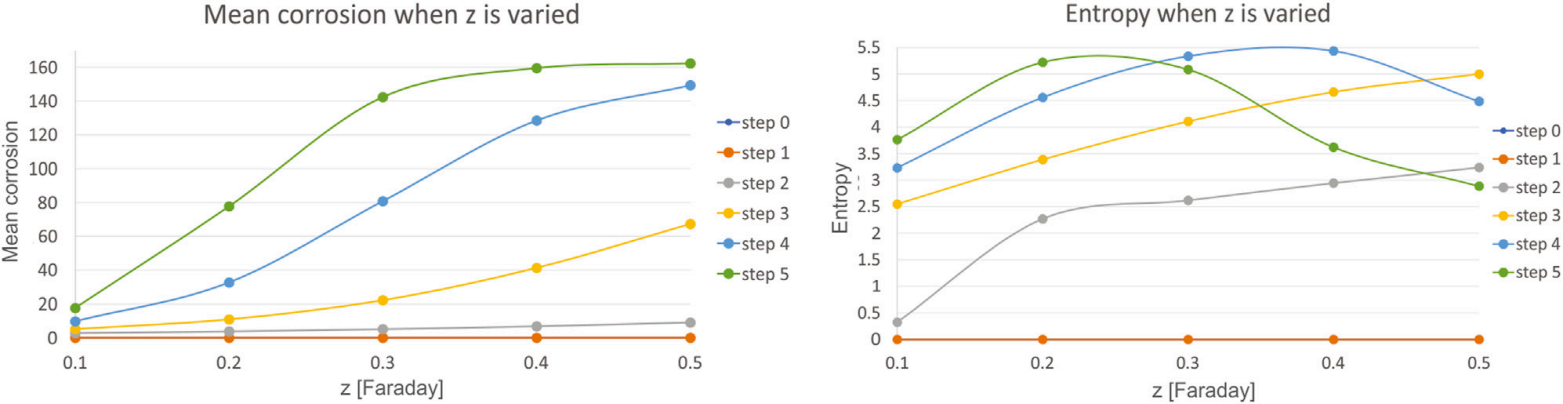
Results for mean corrosion **(left)** and entropy **(right)** when charge of the reaction species z is varied.

Sixth, we have investigated the influence of charge of the reaction species z, which is varied in the range of 0.1–0.5, with a step of 0.1. Mean corrosion and entropy for the variation of this parameter are presented in Figure 9.

The charge of the reaction species z has almost the same effect on the mean corrosion as the reaction species C, indicating high level of influence, e.g. very high level of corrosion is achieved with small increase of parameter reaction species z.

### Experimental Results

**FIGURE 10:**
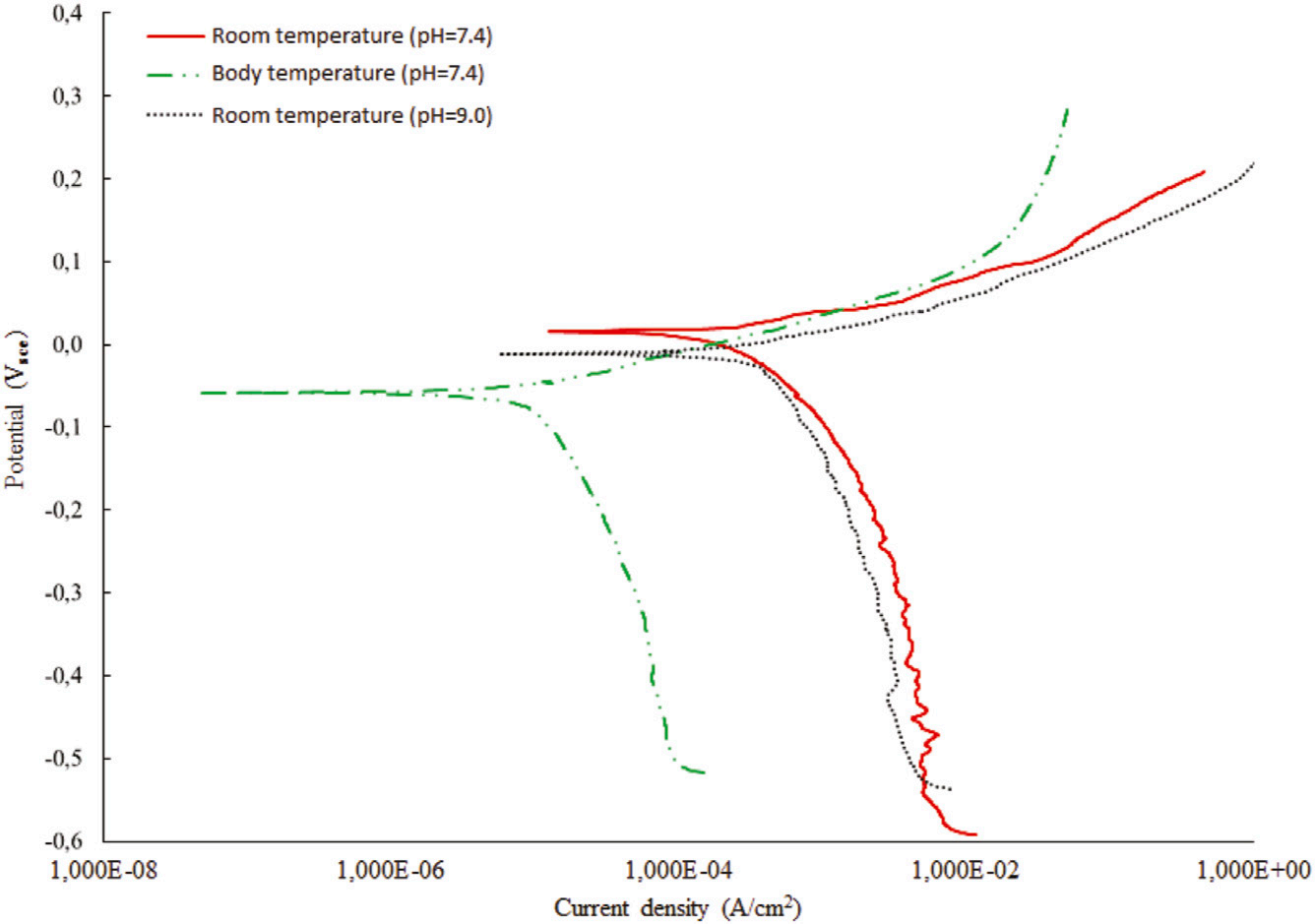
Potentiodynamic polarization measurements of NiTi samples.

**FIGURE 11:**
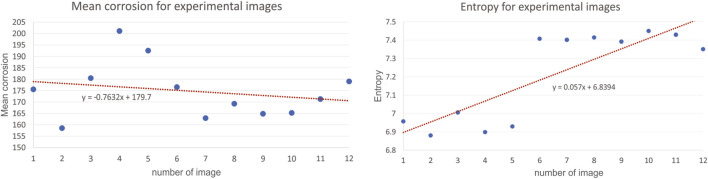
Results for mean corrosion **(left)** and entropy **(right)** for experimental images.

Corrosion experiments were performed in SBF solution with different temperature and pH parameters. The first test was performed with two different pH value as explained in Table 6. The corrosion current density (I_corr_), the corrosion potential (E_corr_) and corrosion rates were found by using the Tafel fit method (Mansfeld 1976). All corrosion measurements were performed in duplicates. The results showed that the corrosion resistance performance degraded with the increased pH value of the solution (Figure 10). On the other hand, Table 7 shows the test results that obtained at room temperature and body temperature for constant pH value, 7.4. It was found that there is no significant difference found for different temperatures, which is in accordance with the simulation results as well, thus these measurements agreed with the simulation results that were given in the previous sections (Figure 11).

**TABLE 7:** Potentiodynamic polarization results of samples at different temperature (pH: 7.4).

Temperature (°C)	Corrosion rate (mpy)	Error
Room temperature	774.2	0.4
Body temperature	784.9	0.1

### Comparison Between the Simulation and Experimental Results

Numerical simulation results in terms of corrosion investigation are difficult to confirm experimentally since it requires the use of specialized equipment. The potential difference, concentration of the reaction species, diffusivity of the reaction species, charge of the reaction species changes throughout the corrosion process were not monitored due to the nature of the electro-chemical process. Also, large number of experiments regarding variations of pH and temperature are time consuming to perform. In such circumstances, our simulation findings should be useful in better understanding the spatial influence of various factors on the material surface.

**FIGURE 12:**
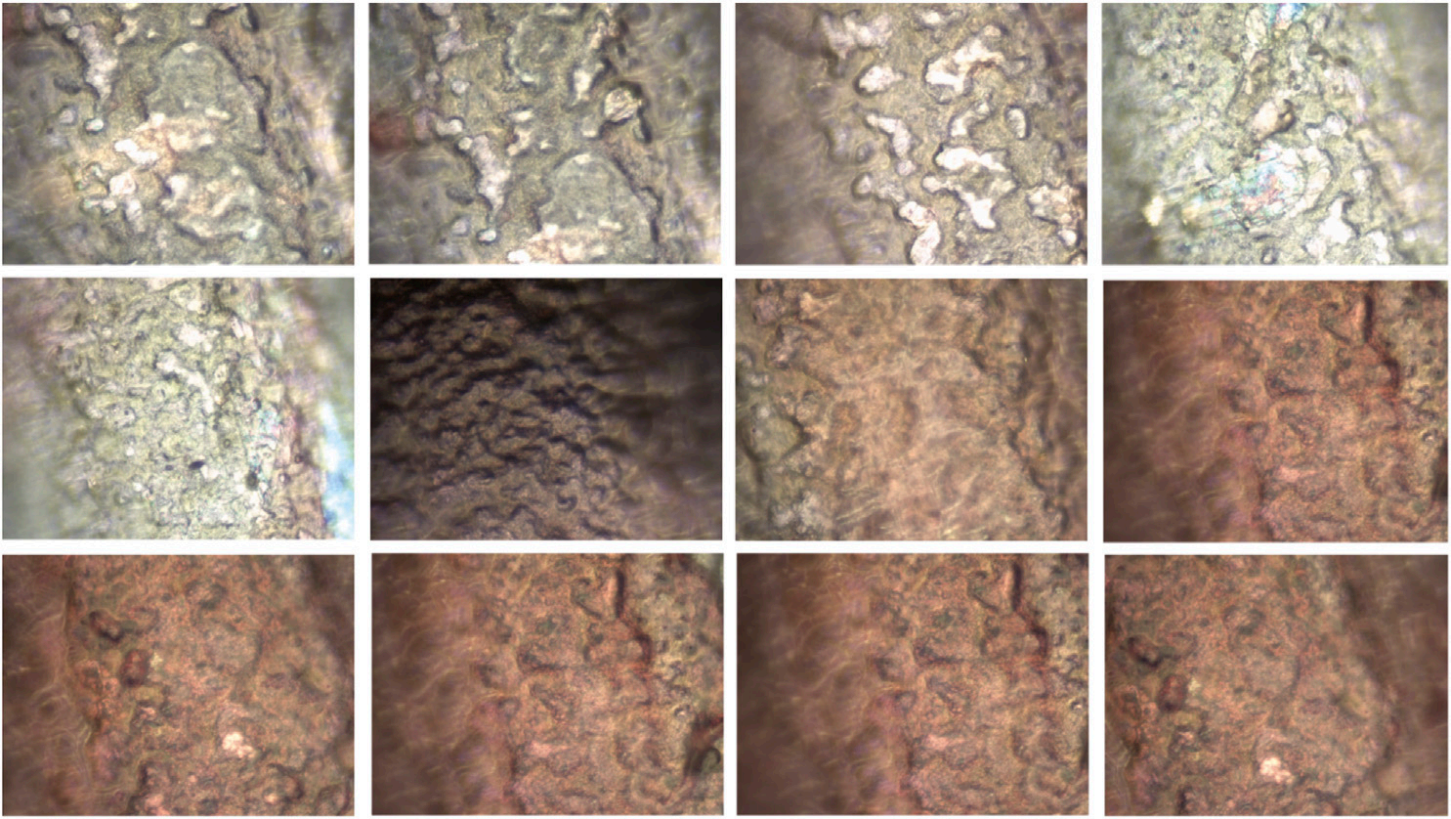
Sets of optical microscope images with 50x magnification.

To validate simulation model with the experimental data, to the certain extent possible, we extracted same features from both the corrosion simulation images and experimental images, and then performed a feature analysis to compare the results. We have investigated the analysis of several metrics, used to assess the corrosion in numerical simulations, also for experimentally available images. To illustrate the calculation for the same metrics for the images coming from experiments, we are presenting here the results for 12 images only as shown in Figure 12. All images were taken by optical microscope. Mean corrosion and entropy for experimental images are presented are Figure 11. Results for all the rest images were similar, meaning the same conclusions can be drawn.

The investigation results of a mean corrosion and entropy for one set of experimental images show that the images taken at different positions of the same corroded wire do not change much. Although the data look linear, the attention should be paid to the value of slope coefficient, as it is close to 0, showing that actually the data are more of a constant, which in return means that the methodology for calculating mean corrosion and entropy is adequate in the analysis of images. Theoretically, the data for one experiment should show uniform values around constant line, however, due to the variations of surfaces, conditions, angle of imaging etc., there is some scatter (Pidaparti et al., 2005).

As the values for environmental factors (pH, potential difference, temperature, concentration of the reaction species, diffusivity of the reaction species and charge of the reaction species) in the experiment were not all available, it was not possible to completely compare the experiment and simulations. However, the values for mean corrosion and entropy show the same order of magnitude is achieved in simulations and experiments, indicating that the methodology used in simulations is adequate.

Moreover, review of the literature regarding comparison of the numerical results with other experiments in the literature showed good comparison of proposed methodology with both numerical simulation of other authors, as well as their experimental work. Pidaparti et al. (Pidaparti et al., 2010) showed that the material loss curve has the same trend as mean corrosion in our paper. Additionally, (Pidaparti et al., 2005), showed that in both corrosion simulations and experiments entropy feature follows the trend of increase up to a certain number of simulations and then decreases after that. This finding is the same as in our paper, with the same order of magnitude and maximal value of approximately 7. In contrast, they also showed that extent of corrosion has the trend of sigmoidal function with steepness coefficient larger than 1, meaning that it increases up to certain number of cycles, by which time the whole surface is completely corroded. The same trend and curve shape is observed in our research.

Additional review of the literature has shown that pH change over time in experiments has the influence on corrosion in such a way that higher pH value affects faster corrosion in time. This is also shown by our experiments visible in Table 6. Factor of pH is important in the corrosion resistance of material because hydrogen ions can interact with a material and modify the surface which can affect it’s corrosion resistance (Trépanier and Pelton 2006). The influence of pH in simulations is discussed regarding Figure 4 showing that the greater corrosion is achieved over time. The results for entropy show that after certain threshold, the entropy reduces and converges after the value of pH = 8.8–9, which also corresponds to the fixed value of mean corrosion. This is in correspondence with the findings from (Capoşi et al., 2011) showing that the corrosion rate is higher for the first hours of immersion and after several days the values become stationary. Temperature effects on corrosion have shown both in literature and simulations that there is no major effect on corrosion in the investigated ranges (Trépanier and Pelton 2006).

**FIGURE 13:**
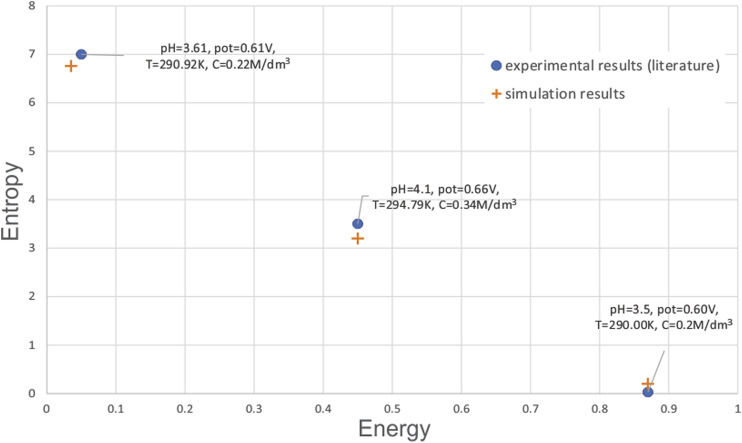
Comparison of simulation results with experimental results from literature.

Since not many papers use the metrics such as entropy and energy, we have compared the results from our simulation with results from (Pidaparti et al., 2005). The comparison is shown in Figure 13. It can be seen that for the given values of environmental factors pH, potential, temperature and concentration of reaction species, there is a good match between the literature experimental findings and simulation from our study. Some small differences can be explained by the fact that not all environmental parameters were given in the referenced paper.

## Conclusion

Proper prediction of the corrosion process is very important when it comes to metallic implants. The body environment is aggressive to metallic biomaterials and can cause corrosion by several different mechanisms. Corroded implants can release into the body species that are allergenic, inflammation inducing, toxic or carcinogenic. Also, developed corrosion process can lead to the mechanical implant failure. The corrosion of the implant affects biocompatibility of the used material. Thus, from the biocompatibility aspect it is desirable to avoid corrosion process as much as possible. Because of these reasons testing of the implants for corrosion process is required.

Even though experimental testing on the biomaterials for corrosion are performed, it is hard to experimentally predict all the situations that can occur in the body. The reasons for incomplete experimental testing are stochastic nature of corrosion process, the need for long-term data collection and different responses of different patients to the same biomaterials.

A great easing of experimental burden in definition of corrosion process can be achieved by computational modelling. There are several advantages of creating computational models. Usage of computers and numerical methods to simulate the process of corrosion will yield to much faster response and collecting of the data in comparison to the corresponding experiments. The computational/*in-silico* models can be easily modified to include more input parameters including disturbance parameters. Finally, *in-silico* models for predicting corrosion process of biomaterials can be beneficial in terms of safety, since number of experiments can be reduced and replaced with numerical simulations (virtual experiments). Even though number of experiments can be greatly reduced with usage of computational models, certain number of experiments has to be performed in order to validate developed computational models.

In this paper the problem of corrosion initialization and progress has been investigated from an *in-silico* point of view using cellular automata model. A discrete dynamic model is implemented to simulate the mechanism of corrosion initialization and its further progress, using the rules that involve electrochemical reactions. Besides, a thorough investigation has been performed to determine which parameters influence the corrosion progress mechanism. The major conclusions of presented *in-silico* model are–1) increase of pH leads to increase of corrosion, 2) higher potential difference increases both the chemical reaction rate and the mass transport rate, which in turn increases the corrosion rate, 3) ambient temperature range around 24–40°C does not influence the corrosion almost at all, and 4) very high level of corrosion is achieved with small increase of parameter reaction species.

Preliminary results from *in-silico* models show that the developed model accurately captures the corrosion progress development in response to changes of different environmental parameters.

The main limitation of the study is that it was not possible to completely validate the *in-silico* model, due to large number of factors influencing the corrosion and experimental investigations are extremely difficult to perform since it requires the use of specialized equipment. Additionally, there is the scatter in the experimental data due to various parameters affecting the corrosion damage process and it was not possible to determine all the values of parameters in experiments. However, preliminary steps taken towards complete validation include comparison of features extracted from the simulation images with images from experiments and comparison with literature. Results from simulations have shown a good match with the features from experiments and literature, and therefore after additional validation, this model can support the prediction of long-term corrosion.

In order to ease all future investigations of the influence of different parameters, as well as to compare experiments with simulations, a platform with the online model was created. That platform already proved itself to be a very useful online tool for model testing in a sense of easiness of environment factors optimization with a goal to postpone the material corrosion.

## Data Availability

The raw data supporting the conclusion of this article will be made available by the authors, without undue reservation.
