# Supplementary material for: An In-Silico Corrosion Model for Biomedical Applications for Coupling With In-Vitro Biocompatibility Tests for Estimation of Long-Term Effects
Source: Front Bioeng Biotechnol. 2021 Sep 7;9:718026. doi: 10.3389/fbioe.2021.718026 (PMC8452857; doi:10.3389/fbioe.2021.718026)
Supplement: Supplementary file 1 [file Presentation1.pdf]

Supplementary materials

1 Computational platform

In order to bring the research closer to the end-users, a user-friendly web platform that follows the workflow of two main sections (corrosion model and corrosion experiment) was added to this study. Main sections are *Corrosion model*, which performs the in-silico simulation of the corrosion and *Corrosion Experiment*, which analyses the experimental images.

The web platform is implemented using microservices. There are three endpoints: the first one to run the calculation, the second one to check the status of the calculation, and the third one to download the results. The first one works the longest, and only for as long as it takes to create a calculation/working directory, copy and create the necessary files, and start the calculation. Among other services, we will explain only the one that serves to delete the directory of the finished calculation. It is aimed for the client to call it upon receiving results as that will be considered as a polite behavior of a client. However, the server will not be overwhelmed with directories of obsolete calculations because there is a special background service that deletes them. On the other side, the web interface that is currently presented below in the text and reflects the current needs of identified users. However, if there happens to be a request to create a client desktop or mobile application, that won't require any additional changes to the server. When it comes to containerization, it will be considered only in case the current hardware capacities won't be enough to fit scalability issues so the cloud becomes a right option.

The reasons behind the decision for the current server-side implementation are mainly that the user is not dependent of the processing software installation. This means that no Matlab/Matlab Runtime is necessary for the user to be downloaded, and the user does not need to have any previous programming knowledge, as web services are arranged in a user-friendly environment/interface. It can be accessed and run from any browser, so no installation of additional application is required. Such server-side implementation concept allows the development of computational pipelines for remote mathematical analysis of the obtained experimental data and creating of large database that can be further utilized. This application is part of the larger platform, developed during the PANBioRA project[1], that aims to put demanding computational models (including FEM, CA and other) under one umbrella. Therefore, although this specific corrosion model is not computationally expensive, it is part of the platform related to web services for complex biomaterial analysis.

1.1 Server-side implementation

Since the calculation was to be performed on a server, it was necessary to provide appropriate web services to run it, check the current status of calculation and finally, download the results. What was especially challenging regarding this case is that the calculation itself can take an unusually long time to run, compared to classical web applications. Namely, the calculations of such kind using Cellular Automata can last approximately up to 15 minutes, so the client has to be aware of that fact and be regularly informed about whether the calculation is still active or not.

---

[1] https://www.panbiora.eu/

On every client request for a new calculation, a server creates separate directory to handle it. In there, it copies the calculation application itself, creates config files from request parameters and then runs it as a completely independent process. Then, it returns to the client an ID of the process so it will be able to periodically check the status of the calculation and eventually get the results.

The workflow at the server side is presented in **Figure S1**. Note that it is essentially one picture but due to its size, it's being split into two parts (left and right).

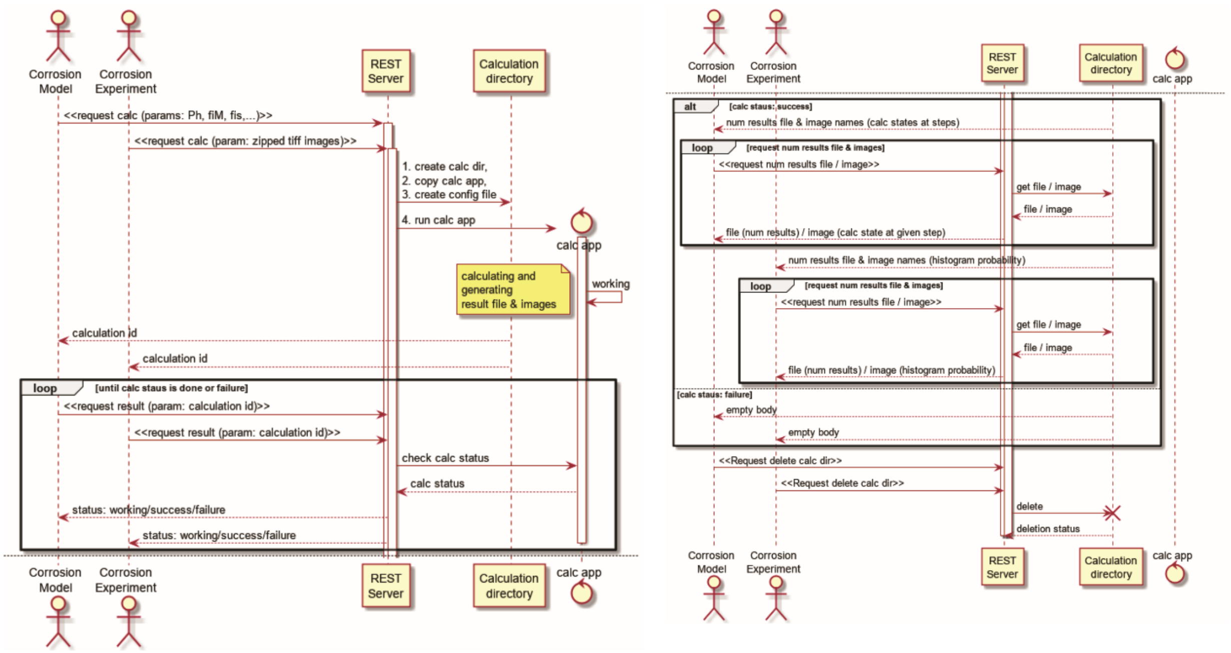

Figure S1. Sequence diagram of the server-side workflow

Additionally, in order to ensure the overall robustness of the application, the validation of input parameters is performed, both on the client and the server side, while detecting and processing all possible errors that may occur either due to the server calculation itself or during network communication.

1.2 Client-side implementation

On the other side, the client part is realized in the form of a webform that enables sending request parameters for a new calculation, checking its current status and finally getting results.

When the corrosion model is selected, a corresponding part of the whole web form will be highlighted so the user will be able to enter required parameters or simply let the application chose default values (**Figure S2**). Each parameter is described using the tooltip on the right side, next to the parameter unit. Acceptable ranges, as well as default values are also described in the tooltip. If only the in-silico model is used, the user should enter the input parameters for simulation, after which the model will be started, images for each time step presented to the user in the form of a PNG image and in each time step, values for all statistical metrics are calculated. It should be emphasized that platform displays images and calculates statistical measures for every t=20*n time

steps to ensure the corrosion progress is visible. These measures are calculated using image processing techniques and are all based on pixel intensities and image histogram probabilities. More detailed explanation of how each measure is calculated in image processing is given in Table 4.

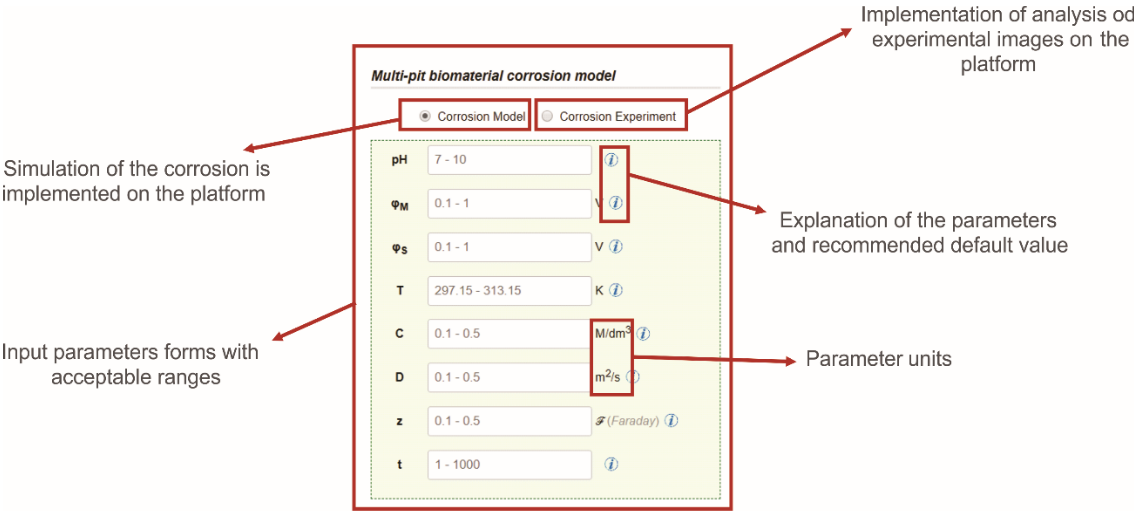

Figure S2. User interface for in silico simulation of biomaterial corrosion process

On corrosion experiment selection, another part of the user interface will be highlighted and become active (**Figure S3**). Tooltip on the right side gives explanation of the expected format of the images – a zip file containing TIF images as a result of a single experiment. In case that experimental images are available, they are uploaded and the same statistical measures are calculated for the experimental images.

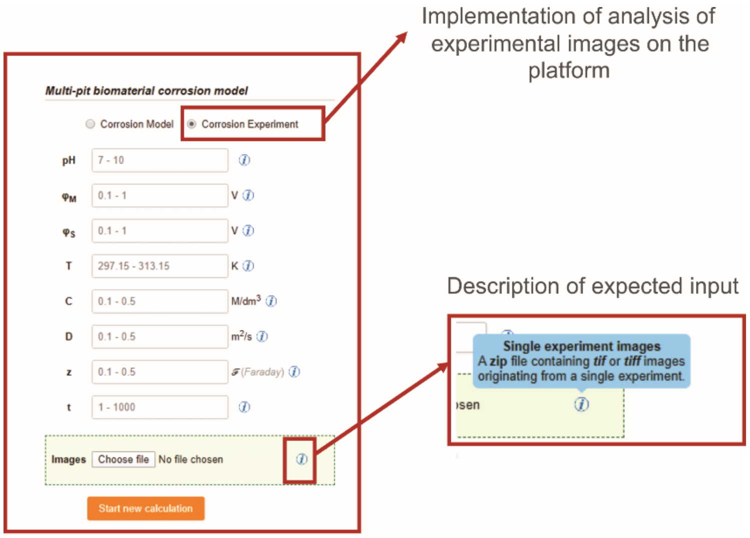

Figure S3. User interface for analysis of experimental images during biomaterial corrosion process

After either the corrosion model or corrosion experiment have been started, the results are displayed in the form of different images and numerical results, as was previously explained. Once the results are available, a *.txt* file will be generated.

The proposed methodology is performed in such a way that the user can run independently in silico simulations of corrosion and analysis of such obtained images, and analysis of experimental images. This is meaningful as not every user would have the possibility to perform experiments, but may be interested in silico model. If the environmental condition values given in Table 3 are available from the experiment, the user can perform both in silico simulations and the same analysis for the experimental images, in order to compare the results.

2 Results visualized on the platform

In the case of corrosion simulation model, resulting images are displayed on the web platform together with the histogram probability of the last state. Each figure can be saved as *.png* for offline analysis (**Figure S4 – right side**). Numerical results are available for download in the results section bellow the display of the used values for input parameters in that calculation (**Figure S4 – left side**).

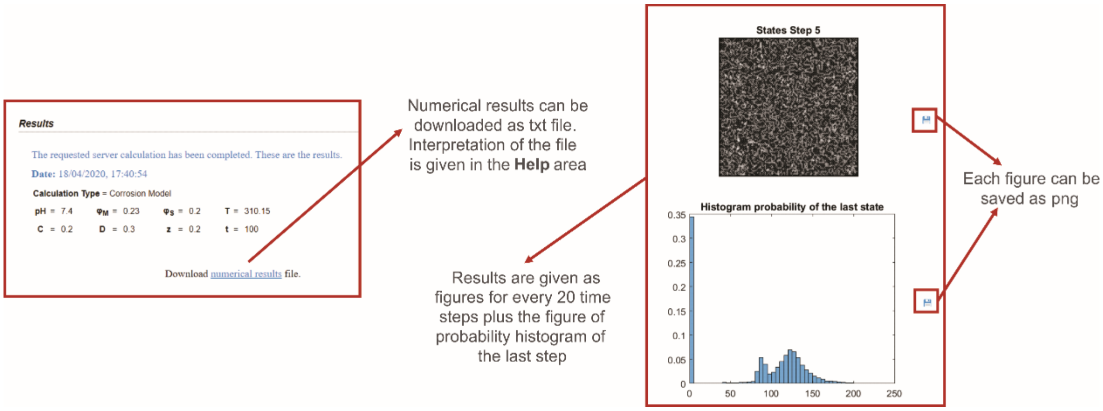

Figure S4. Results section for corrosion simulation model on the platform

In the case of corrosion experiment calculation, the uploaded images are analyzed and numerical results are available for download in the results section bellow the display of the name of the uploaded zip file (**Figure S5 – upper side**). Images of histogram probabilities for each experimental image in the zip file are displayed and available to be saved as *.png* for offline analysis (**Figure S5 – bottom side**).

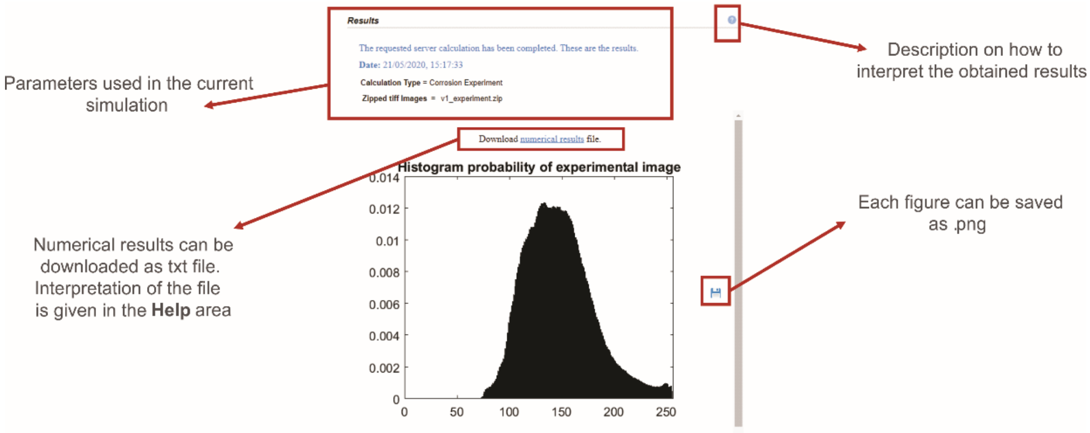

Figure S5. Results section for corrosion experiment calculation
